# Epigenetic assays for chemical biology and drug discovery

**DOI:** 10.1186/s13148-017-0342-6

**Published:** 2017-04-21

**Authors:** Sheraz Gul

**Affiliations:** Fraunhofer Institute for Molecular Biology and Applied Ecology - ScreeningPort, Schnackenburgallee 114, 22525 Hamburg, Germany

**Keywords:** Assay development, Bromodomain, Chemical biology, Chemical probe, Drug discovery, High throughput screening, Histone acetyltransferase, Histone deacetylase, Histone methyltransferase, Demethylase

## Abstract

The implication of epigenetic abnormalities in many diseases and the approval of a number of compounds that modulate specific epigenetic targets in a therapeutically relevant manner in cancer specifically confirms that some of these targets are druggable by small molecules. Furthermore, a number of compounds are currently in clinical trials for other diseases including cardiovascular, neurological and metabolic disorders. Despite these advances, the approved treatments for cancer only extend progression-free survival for a relatively short time and being associated with significant side effects. The current clinical trials involving the next generation of epigenetic drugs may address the disadvantages of the currently approved epigenetic drugs.

The identification of chemical starting points of many drugs often makes use of screening in vitro assays against libraries of synthetic or natural products. These assays can be biochemical (using purified protein) or cell-based (using for example, genetically modified, cancer cell lines or primary cells) and performed in microtiter plates, thus enabling a large number of samples to be tested. A considerable number of such assays are available to monitor epigenetic target activity, and this review provides an overview of drug discovery and chemical biology and describes assays that monitor activities of histone deacetylase, lysine-specific demethylase, histone methyltransferase, histone acetyltransferase and bromodomain. It is of critical importance that an appropriate assay is developed and comprehensively validated for a given drug target prior to screening in order to improve the probability of the compound progressing in the drug discovery value chain.

## Background

Chemical biology makes use of chemistry to understand biological processes and this overlaps significantly with drug discovery, especially when the latter focusses on small molecules [1]. Chemical biology could also be considered to have a more basic research focus in that the research is largely directed towards understanding fundamental biological processes with small molecules being used as tools to facilitate this [2, 3]. This approach is complementary to molecular biological methods where mutations of residues in proteins are utilized to determine the roles they play in biological processes. In many cases, the small molecules in chemical biology can also serve as starting points for drug discovery and this is exemplified by the concept of “chemical probe” [4–8]. The key attributes of a “chemical probe” includes defined mechanism of action, appropriate selectivity, often being freely available (both the physical compound and activity data), possessing drug-like properties and being associated with a reliable structure-activity relationship (SAR). These attributes are also relevant for lead compounds, clinical candidate molecules and drugs, but will also have additional attributes such as intellectual property rights, human bioavailability, and appropriate physicochemical and pharmaceutical properties.

Drug discovery is a high risk, expensive and lengthy process, typically lasting 10 years with defined phases [9]. The pre-clinical stage of drug discovery, sometimes also referred to the gene-to-candidate phase, can span a period of 5 years before the compound is suitable for human clinical trials. During this stage, a target deemed worthy of therapeutic intervention is identified and subsequently a biological reagent (usually purified protein or cell line) is prepared that contains the target of interest. In the case of small-molecule drug discovery, this biological reagent would then be utilized to develop an appropriate assay for monitoring target activity and screened against libraries of small molecules (hundreds to millions of compounds) [10–12]. Evaluation of the active compounds from the screening campaign (hits) with freshly synthesized compounds meeting acceptable purity and integrity in a panel of relevant assays would ultimately yield a validated hit list comprising a data package pertaining to the biological activity [13]. Each validated hit series would then be annotated with additional data such as the Lipinski rule of five [(i) molecular weight less than 500, (ii) logP, a partition coefficient measuring hydrophobicity less than five, (iii) no more than five hydrogen bond donors and (iv) no more than 10 hydrogen bond acceptors]. Bearing in mind the high attrition of drug discovery, more than one of the most promising validated hit series would be progressed to the hit-to-lead (H2L) phase [14]. Several iterative rounds of synthesis would enable the optimisation of the potency of compounds against the target of interest to the desired criteria for a lead series (typically in the sub-micromolar range) whilst retaining an appropriate selectivity profile. Additional information required when selecting the final lead series will include demonstrable and acceptable SAR, off-target selectivity profile, toxicity, physicochemical profile, solubility and stability in aqueous solution and human plasma, in vivo pharmacokinetics, Absorption, Distribution, Metabolism and Excretion (ADME) properties, patentability and competitor activity. Further significant optimisation of a compound within the lead series would result in the generation of a pre-clinical candidate compound and, upon approval by the relevant regulatory organizations, can enter human clinical trials [9].

In the post-Human Genome Project era [15], target-based drug discovery accelerated considerably and is fittingly illustrated by the kinase target class [16]. A consequence of target-based drug discovery has been the multitude of assays being available for most target classes and the remainder of this article focusses on general concepts of assay development with a specific focus on screening compatible assays for epigenetics targets, and Table 1 provides a summary of the assays. Many of the epigenetic assays reported in the literature and referred to herein make use of commercial extensively validated kits. Where possible, original references are cited that would enable an understanding of the rationale for the development of epigenetic assays and their utilization in a variety of research activities.

**Table 1:** Screening compatible epigenetic assays

Enzyme	Assay format	Key features of the assay	References
Histone deacetylase (HDAC)	Chemiluminescent (AlphaLISA®)	• Assay reported in literature and commercial validated assay kit • Substrate: histone proteins • Detection: H3-K9(Ac) or H3-K27(Ac) • High sensitivity • High throughput functional assay	122–124
	Chromatin immunoprecipitation	• Assay reported in literature using specific commercial reagents • Detection: Ac-H3 • High sensitivity • Low throughput assay	125
	Colorimetric (*Color de Lys*®)	• Commercial validated assay kit • Substrate: peptide containing ε-acetylated lysine • Detection: deacetylated peptide via coupled assay • Low sensitivity • Low/Medium throughput functional assay • Prone to optical interference with compounds	126
	Fluorometric (Fluor de Lys®)	• Assay reported in literature and commercial validated assay kit • Substrate: peptide containing ε-acetylated lysine • Detection: deacetylated peptide via coupled assay • Medium/High sensitivity • Medium/High throughput functional assay	127, 128
	Luminescence (HDAC-Glo™ I/II)	• Assay reported in literature and commercial validated assay kit • Substrate: peptide containing ε-acetylated lysine • Detection: deacetylated peptide via coupled assay • High sensitivity • Medium/High throughput functional assay	131, 132
	TR-FRET (LANCE® Ultra)	• Uses specific commercial reagents • Substrate: biotinylated Histone H3-K27(Ac) or Histone H3-K9(Ac) peptide • Detection: H3-K9(Ac) or H3-K27(Ac) • High sensitivity • High throughput functional assay	133
	TR-FRET (LanthaScreen™)	• Assay reported in literature and commercial validated assay kit • Ligand: Alexa Fluor® 647-labelled HDAC inhibitor as a tracer • Detection: displacement of Alexa Fluor® 647-labelled HDAC inhibitor • High sensitivity • High throughput binding assay	134
Demethylase (LSD and Jumonji C domain-containing histone demethylase)	Colorimetric	• Assay reported in literature using specific commercial reagents • Substrates: Histone H3-K4 peptide • Detection: H_2_O_2_ or H3-K4 via coupled assay • Low sensitivity • Low throughput functional assay • Prone to optical interference with compounds	139–142
	Colorimetric (Epigenase™)	• Commercial validated assay kit • Substrates: Histone H3-K4(Me_2_) or dimethylated Histone H3-K4 peptide • Detection: H_2_O_2_ via coupled assay • Low sensitivity • Low throughput functional assay • Requires wash steps • Prone to optical interference with compounds	143
	Fluorescence polarization	• Assay reported in literature using methylstat^fluor^ tracer • Ligand: methylstat^fluor^ tracer • Detection: displacement of methylstat^fluor^ tracer • High sensitivity • High throughput binding assay	144–145
	Fluorometric	• Commercial validated assay kit • Substrate: Histone H3-K4(Me_2_) peptide • Detection: H_2_O_2_ via coupled assay • Low sensitivity • Low throughput functional assay • Requires wash steps • Prone to optical interference with compounds	146–147
	Fluorometric	• Commercial validated assay kit • Substrate: Histone H3-K4(Me_2_) protein • Detection: formaldehyde via coupled assay • Low sensitivity • Low throughput functional assay • Requires wash steps • Prone to optical interference with compounds	148–149
	High content screening	• Assay reported in literature using specific commercial reagents • Substrate: Histone H3-K27(Me)_3_ peptide • Detection: H3-K27(Me)_3_ • Medium sensitivity • Medium throughput functional assay	150
	Mass spectrometry	• Assay reported in literature using specific commercial reagents • Substrate: dimethylated peptides • Detection: demethylated products • Medium/High sensitivity • Low throughput functional assay • No optical interference from compounds	151, 152
	Radioactive	• Assay reported in literature using specific commercial reagents • Substrate: ^3^H-labelled methylated histone • Detection: ^3^H-formaldehyde via coupled functional assay • No optical interference from compounds • Radioactive waste	153, 154
	TR-FRET (LANCE® Ultra)	• Commercial validated assay kit • Substrate: biotinylated Histone H3-K4(Me) peptide • Detection: H3-K4 • High sensitivity • High throughput functional assay	155
Histone methyltransferase (HMT)	Chemiluminescent (AlphaLISA®)	• Commercial validated assay kit • Substrate: Histone H3-K79(Me_2_) protein • Detection: H3-K79(Me_2_) • High sensitivity • High throughput functional assay	161
	Fluorescence polarization	• Assay reported in literature using specific commercial reagents • Substrate: protein or peptide • Detection: displacement of labelled-AMP in coupled assay • High sensitivity • High throughput binding assay	162
	Fluorometric	• Assay reported in literature using specific commercial reagents • Substrate: Histone H3 peptide • Detection: homocysteine via coupled assay • High sensitivity • High throughput functional assay	163–165
	High content screening	• Assay reported in literature using specific commercial reagents • Substrate: Histone H3-K27(Me)_3_ • Detection: H3-K27(Me)_3_ • Medium sensitivity • Medium throughput functional assay	166
	Luminescence	• Assay reported in literature using specific commercial reagents • Substrate: protein or peptide • Detection: complex coupled assay • High sensitivity • High throughput functional assay	167
	Radiometric	• Assay reported in literature using specific commercial reagents • Substrate: biotinylated Histone H3-K9 peptide • Detection: ^3^H-incorporated into peptide • No optical interference from compounds • Radioactive waste	168–170
Histone acetyltransferase (HAT)	Colorimetric	• Commercial validated assay kit • Substrate: proprietary peptide • Detection: NADH via coupled assay • Low sensitivity • Low/Medium throughput functional assay • Prone to optical interference with compounds	174
	ELISA	• Commercial validated assay kit • Substrate: histone • Detection: H3-K4(Ac) via coupled assay • Low/Medium sensitivity • Requires wash steps • Medium throughput functional assay	175
	Fluorometric	• Assay reported in literature using specific commercial reagents • Substrate: Histone H3 or Histone H4 peptide • Detection: CoA-SH via coupled assay • High sensitivity • High throughput functional assay • Prone to optical interference with compounds	176
	Fluorometric	• Commercial validated assay kit • Substrate: Histone protein • Detection: CoA-SH via coupled assay • High sensitivity • High throughput functional assay	177
	Microfluidic mobility shift	• Assay reported in literature using specific commercial reagents • Substrate: fluorolabelled Histone H3 or Histone H4 peptide • Detection: charge difference of substrate/product • High sensitivity • Medium throughput functional assay • No optical interference from compounds	178
	Radiometric	• Assay reported in literature using specific commercial reagents • Substrate: biotinylated Histone H4 peptide or histone protein • Detection: ^3^H-incorporated into peptide or histone • High sensitivity • Low throughput functional assay • No optical interference from compounds • Radioactive waste	179–181
	TR-FRET(LANCE® Ultra)	• Commercial validated assay kit • Substrate: biotinylated Histone H3-K9 peptide • Detection: H3-K9(Ac) • High sensitivity • High throughput binding assay	182
Bromodomain	Chemiluminescent (AlphaScreen™)	• Assay reported in literature using specific commercial reagents • Substrate: biotinylated Histone H4-K5(Ac) • Detection: presence of BRD4/peptide complex • High sensitivity • High throughput binding assay	188, 189
	Differential Scanning Fluorometry (BromoMELT™)	• Assay reported in literature and commercial validated assay kit • Substrate: BRD4 • Detection: *T*_m_ of BRD4/SYPRO Orange complex • Low/Medium sensitivity • Medium throughput binding assay • No optical interference from compounds	190, 191
	Fluorescence polarization	• Assay reported in literature using specific commercial reagents • Substrate: fluorescent BODIPY labelled tracer • Detection: BRD4/BODIPY labelled tracer complex • Medium sensitivity • Medium throughput binding assay	190
	TR-FRET	• Commercial validated assay kit • Substrate: biotinylated peptide • Detection: BRD4/biotinylated peptide complex • High sensitivity • High throughput binding assay	192

## Assay development, high throughput and high content screening in pre-clinical drug discovery

Assays that are screened against libraries of compounds to identify chemical starting points in the early stages of drug discovery can be classified as being biochemical or cell-based in nature. The exact assay that is utilized in a screen is decided upon a case-by-case basis after taking into account a number of factors such as provision of reagents, throughput, cost, and many others that have been discussed extensively in the literature [17]. The biochemical target-based (reductionist) approach was largely adopted in the post-Human Genome Project era where specific genes were identified and cloned and the corresponding proteins expressed in sufficient quantity with acceptable activity for screening [18]. This was a marked shift from earlier cell-based assays where modulation of specific targets did not occur, but instead relevant cellular phenotypic responses were measured [19, 20]. Significant effort has been expended to mimic these physiologically relevant cell-based systems with a significantly higher throughput [21] and advances have been made using a variety of these and subsequently deployed in cancer drug discovery in particular [22–24] as well as being expanded to areas such as predictive toxicology [25].

**Fig. 1:**
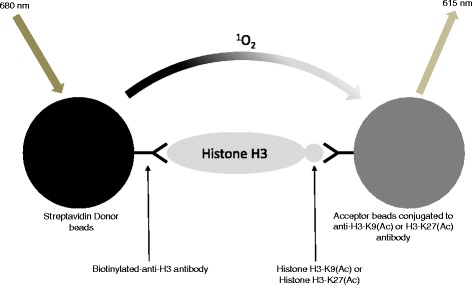
*AlphaLISA*® histone deacetylate assay that detects Histone H3-K9(Ac) or Histone H3-K27(Ac). The acetylated histones are detected using a biotinylated anti-H3 antibody and AlphaLISA®-acceptor beads conjugated specific to the acetylated lysine. Streptavidin-donor beads then capture the biotinylated antibody, bringing the acceptor and donor beads into proximity. Upon laser irradiation of the donor beads at 680 nm, short-lived singlet oxygen molecules produced by the donor beads can reach the acceptor beads in proximity to generate an amplified chemiluminescent signal at 615 nm

**Fig. 2:**
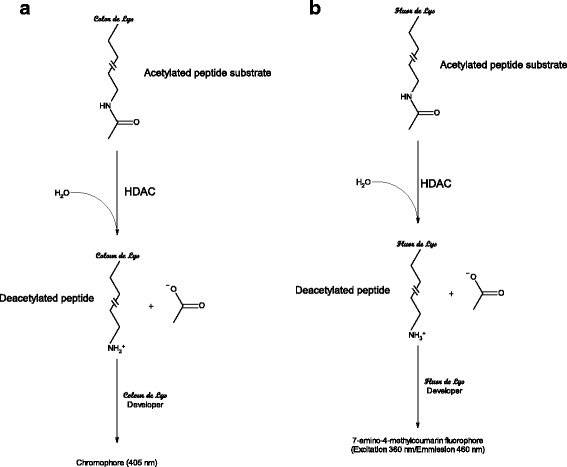
**a** Colorimetric coupled histone deacetylate assay that makes use of a chromogenic peptide substrate (proprietary *Color de Lys*® Substrate) containing a ε-acetylated lysine residue. When an HDAC enzyme acts upon the substrate and the sidechain of a ε-acetylated lysine residue is deacetylated, it becomes susceptible to further degradation by an enzyme in the developer reagent (proprietary *Color de Lys*® Developer). The action of the enzyme within the developer reagent results in the release of a chromophore detected by measuring the absorbance of the reaction at 405 nm. **b** Fluorometric coupled histone deacetylate assay that makes use of a fluorogenic peptide substrate (proprietary *Fluor de Lys*® Substrate) containing a ε-acetylated lysine residue. When an HDAC enzyme acts upon the substrate and the sidechain of a ε-acetylated lysine residue is deacetylated, it becomes susceptible to further degradation by an enzyme in the developer reagent (proprietary *Fluor de Lys*® Developer) resulting in the release of 7-amino-4-methylcoumarin fluorophore which undergoes excitation at 360 nm and emits at 460 nm

**Fig. 3:**
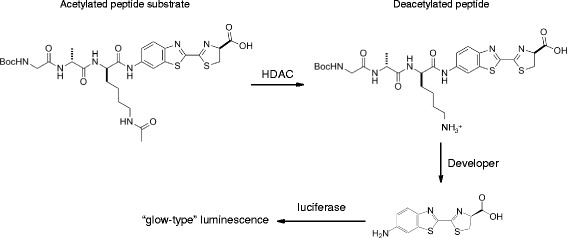
Luminescence coupled histone deacetylate assay that makes use of specific amino-luciferin labelled ε-acetylated lysine peptide substrates for HDAC Class I/II enzymes. When the substrate undergoes deacetylation by the HDAC enzyme, the product becomes susceptible to the Developer reagent and results in the release of amino-luciferin. This amino-luciferin is the substrate for a luciferase enzyme (also in the Developer reagent) and yields a glow-type luminescence

**Fig. 4:**
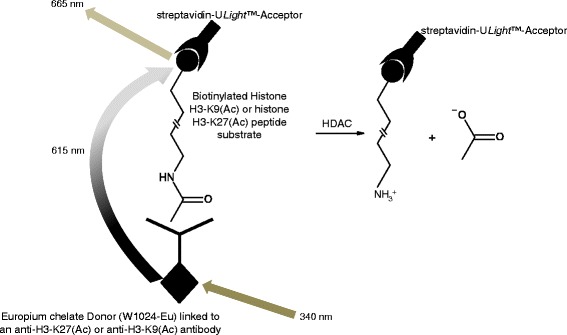
Time-resolved fluorescence resonance energy transfer histone deacetylase assay. A signal is generated when the deacetylated peptides are captured by the Europium-labelled antibody donor and streptavidin-U*Light*™-acceptor thus bringing the Europium-donor and U*Light*™-acceptor molecules into close proximity. Upon irradiation at 340 nm, the energy from the Europium-donor is transferred to the U*Light*™-acceptor, which, in turn, generates a signal at 665 nm

For any given protein target class, a variety of fully validated screening compatible assay kits are commercially available. These offer the potential to reduce cycle times significantly for hit identification and beyond. Alternatively, it may be possible to exploit specific commercial reagents to build de novo assays and this isrelevant when investigating newly identified proteins and their substrates. Where appropriate, schematic representations of assays are provided (Figs. 1, 2, 3, and 4). The ultimate decision as to which assay to use in a screening campaign is usually considered on a case-by-case basis when initiating a drug discovery project since all assays have specific advantages and disadvantages. For example, in the case of the protein kinases, biochemical assays are often utilized and more than 20 of such assays are commercially available [26, 27] whereas in the case of G-protein-coupled receptors, cell-based assays are more commonly employed [28–32]. It is prudent to develop a panel of assays with different readout modes, as these are suitable for the hit validation stage thus allowing confirmation as to whether the activities of compounds translate to more than one assay format thereby adding confidence that they are not assay artefacts [33–36]. This is important as it is now known that assays that make use of specific tagged proteins in the AlphaScreen™ assay format often yield specific interfering compounds as false positive hits [37, 38]. Assay formats that have enhanced the capabilities relative to phenotypic assays include label-free impedance-based [39, 40] dynamic mass redistribution [41, 42] and multiplex assays [43, 44], and these have been successfully applied in screening against small-molecule libraries. More recent state-of-the-art screening compatible assays use three-dimensional spheroids that offer the potential to represent the microenvironment of cells in the body [45].

The pre-requisites for high throughput screening (HTS) are access to a suitable assay as briefly described above and a suitable compound library. Compound libraries are usually stored in pure DMSO at concentrations between 1 mM and 10 mM as this will allow for a range of final assay concentrations of compound whilst retaining <1% DMSO (*v/v*) in the final assay. The extent of automation when embarking upon an HTS campaign will depend upon the numbers of compounds screened and it would be reasonable to screen a compound library composed of a few thousand compounds manually in miniaturized formats (e.g. 384- or 1536-well microtiter plates). However, where >5000 compounds are screened (in 384-well microtiter plates), it would be prudent to use some degree of automation such as stand-alone reagent dispensers or a robotic screening system [46–49]. One way to minimize the consumption of reagents when screening very large numbers of compounds is to miniaturize and parallelize an assay into 1536-well microtiter plates [50]. However, such miniaturization requires the addition of very small volumes of compound stock solutions and technologies such as the contactless acoustic dispenser from Labcyte Inc. makes this possible [51].

High content screening (HCS) is now an established technique that is routinely utilized in chemical biology and drug discovery and has made a significant impact upon understanding the output of phenotypic screening. This is a cell-based approach that can offer a multi-parameter readout detecting simultaneously a multitude of cellular changes that are subsequently attributed to specific targets [52–56]. This approach is particularly relevant in epigenetics as the discovery of Romidepsin and Vorinostat as anti-cancer drugs originates from phenotypic assays [57].

## General concepts underlying the common deployed screening compatible assays Amplified Luminescent Proximity Homogeneous (AlphaLISA^®^ and AlphaScreen^®^) assays

These are proximity-based assays that have successfully been used to study the activity of a wide range of targets [58–61]. The technology requires two bead types, termed donor beads and acceptor beads with the former containing the photosensitizer phthalocyanine, which converts ambient oxygen to an excited and reactive singlet oxygen upon illumination at 680 nm. This reactive singlet oxygen can diffuse approximately 200 nm in solution and has a half-life of 4 μs. If an acceptor bead is within that distance, energy is transferred from the singlet oxygen to thioxene derivatives within the acceptor bead, resulting in light production at 520–620 nm (AlphaScreen®) or at 615 nm (AlphaLISA®) [62]. These assays do not require wash steps unlike in a standard ELISA, see Fig. 1.

### Colorimetric assays

These rely upon the difference in the electronic absorption spectrum of the substrate and product of a reaction. Chromogenic substrates are composed of organic molecules that contain a conjugated system, i.e. a delocalized π-bond system which is usually attributed to alternating single and double bonds. When chromophores absorb ultraviolet (UV) and visible radiation, their electrons undergo excitation from their ground state to excited state and the wavelength of UV or visible light (approximately 200–800 nm) absorbed depends largely on the extent of conjugation, such that the greater the degree of conjugation within the chromophore, the longer the wavelength of light will be absorbed [63, 64]. In some cases, both the substrate and product will absorb light and it will be necessary to monitor the formation of product where the absorption of the substrate does not change. Additionally, the optimal wavelength at which product formation can be detected should be determined after collecting the absorption of pure samples of substrate and product. When the natural substrate is itself chromogenic, this offers the potential to monitor the activity of an enzyme without the need for a synthetic chromogenic substrate. Thus obviating the effects of steric hindrance by an artificial chromophore in the molecule that can interfere with binding within the active centre of the enzyme and potentially confound the identification of substrate competitive compounds. Despite the successful use of colorimetric assays in screening, they are no longer the preferred option and have largely been replaced by alternative assay formats such as fluorescence-based methods [65, 66]. This has been driven by a number of reasons such as colorimetric assays being relatively insensitive, often requiring substantial concentrations of product (typically low micromolar) to be generated for adequate detection. Colorimetric assays are also particularly prone to optical interference due to coloured compounds which are commonly found in small-molecule libraries. These optically interfering compounds are likely to result in many of these being identified as apparent hits in a small-molecule screening campaign but subsequently shown not to be genuine modulators of the activity of the target protein [34, 37, 38, 67–69]. These false positives need to be identified and removed prior to the progression of compounds for drug discovery purposes. One strategy to reduce the number of apparent hits being overrepresented with optically interfering compounds is to determine the activity of the target protein in the presence of compound in kinetic mode; however, this will reduce the throughput of the assay [70].

### Differential scanning fluorimetry assays

This technique makes use of dyes that are fluorescent when present in a non-polar environment such as hydrophobic sites of unfolded proteins relative to aqueous solution (in the case of unfolded proteins) where their fluorescence is quenched [71]. When low *M*_r_ ligands bind and stabilize proteins, the temperature at which this complex unfolds will be raised and this can be quantified from a fluorescence–temperature plot, with the midpoint of the protein unfolding transition defined as the *T*_m_ (melting temperature), reflecting the potency of the low *M*_r_ ligand towards the protein [72–75].

### Enzyme-linked immunosorbent assay (ELISA)

This technique is used in a variety of industries including diagnostics and quality-control checks [76]. In most cases an ELISA involves an antigen being immobilized to a surface that is capable of capturing a molecule that resembles the antigen. Subsequent to a series of wash steps to remove non-specifically bound proteins, a secondary antibody is applied that is linked to an enzyme and the enzyme substrate is added that yields a signal, usually colorimetric or fluorometric [77–79]. The major drawback of ELISA from a screening perspective is their non-homogenous nature and the requirement for wash steps [80].

### Fluorescence polarization assays

This technique relies upon a change in the hydrodynamic radius of a fluorescent entity (when bound to a protein and free in solution) that alters its hydrodynamic radius [81–83]. Most of these assays are based on an indirect measurement of the size change of a protein and fluorescently labelled ligand. A requirement for this technique is the easy conjugation of a fluorophore to a relevant molecular entity. Binding of this ligand would result in a relatively high fluorescence polarization signal. Its displacement from the target by a competitor molecule would lead to a decrease in the fluorescence polarization signal [84, 85].

### Fluorescence intensity assays

These have been used extensively in drug discovery and offer a number of advantages over colorimetric assays such as being significantly more sensitive and less prone to optical interference [86]. There are a large number of fluorophores available that cover most of the electromagnetic spectrum. As a result it is possible to design and synthesize molecules that contain these fluorophores in order to enable them to be employed as tools to develop assays for the investigation of difficult drug targets [87, 88]. Fluorescein has been widely used as a fluorophore in assays but others are available that are associated with reduced compound-mediated interference [89].

### High content screening assays

These make use of a microscope-based method to image cells that can categorize multiple features when using appropriate fluorescent dyes. The image analysis requires algorithms to allow their categorization, especially after exposure to compounds [90, 91]. These assays can be enhanced when working with primary cells and three-diensional cultures that are more physiologically relevant [92].

### Luminescence assays

These use enzymes such as luciferases, and the complementary luciferin photon-emitting substrates [93, 94]. The most widely used enzymes are firefly luciferase, *Renilla luciferase*, and aequorin [95–97]. In the case of firefly luciferase-based assays, beetle luciferin and ATP are combined to form luciferyl-AMP (an enzyme-bound intermediate). This reacts with O_2_ to create oxyluciferin in a high-energy state and a subsequent energy transition to the ground state yielding light.

### Mass spectrometry assays

This has been a longstanding technique and used as a secondary assay due to its relatively low throughput, or for screening modest libraries of compounds [98, 99]. It is a label-free approach as it relies upon the separation and subsequent quantification of typically a substrate and product that has undergone modification that the mass spectrometer is capable of detecting [100]. The current instrument for high throughput mass spectrometry is the Agilent RapidFire that has been used to screen a range of targets with improved quality of the identified hit compounds [101–103].

### Microfluidic mobility shift assays

This electrophoretic technique requires a charge difference to exist between substrate and product and has been most successfully used to investigate the kinase target class [104]. Although these assays have a low throughput, the major advantage they offer is to overcome compound-mediated optical interference, as it is separated during the electrophoretic separation of substrate and product [105]. The assay requires a fluorescently labelled substrate that can be used to detect both the product and any residual substrate [106].

### Radioactive assays

These assays make use of radioisotopes such as ^3^H, ^14^C, ^33^P, ^35^S and ^125^I. Filter-binding assays have historically been extensively adopted to monitor the activity of a wide range of targets [107, 108]. In the case of the neurotransmitter targets, these assays are considered to be the gold standard assay format as they are label-free, highly sensitive and are not prone to interference in a manner that the other optical methods are susceptible [109]. An advancement to these assays is the no-wash scintillation proximity assay (SPA) which makes use of beads embedded with scintillant that can bind the target of interest and give a signal [110–112].

### Time-resolved-Förster resonance energy transfer assays

This is a proximity-based assay that makes use of lanthanide chelate complexes with a long-lived luminescence in comparison with conventional fluorophores. Therefore, enabling the short-lived background interferences that are predominantly compound mediated to be removed [113]. Typically, a TR-FRET signal is generated when a molecule coupled to the Europium-labelled partner (donor) is brought into close proximity to an acceptor molecule, e.g. Allophycocyanin (APC). Upon irradiation at 340 nm, the energy from the Europium-donor is transferred to the acceptor which in turn generates a signal at 665 nm, Fig. 4 [114].

## The histone deacetylase (HDAC) target class and relevant screening compatible assays

The HDAC family of enzymes remove an acetyl group from acetylated lysine residues in appropriate substrates (both histone and non-histone based) [115, 116]. This protein target class has been implicated in cancer [117, 118], cardiovascular [119], inflammatory and infectious diseases [120] and neurodegeneration [121].

### HDAC AlphaLISA^®^ assays

A commercial assay kit is available that detects changes in the levels of Histone H3-acetylated lysine 9 (H3-K9(Ac)) and Histone H3-K27(Ac) in cellular systems [122–124]. The changes in the levels of acetylated histones is performed with histones extracted from cells, followed by addition of a biotinylated anti-H3 antibody and AlphaLISA®-acceptor beads conjugated specific to the acetylated lysine. Streptavidin-donor beads then capture the biotinylated antibody, bringing the acceptor and donor beads into proximity. Upon laser irradiation of the donor beads at 680 nm, short-lived singlet oxygen molecules produced by the donor beads can reach the acceptor beads in proximity to generate an amplified chemiluminescent signal at 615 nm (Fig. 1). Due to the nature of the assay, any changes in the observed signal may be due to reasons other than HDAC inhibition; therefore, care needs to be taken when interpreting the data. As this assay format is essentially proximity based, it can be employed to monitor the modification of a variety of molecules such as appropriately labelled peptide and protein substrates (e.g. biotin, FLAG, GST, His) that undergo acetylation, demethylation, methylation as well as phosphorylation (in the case of kinases) when using an antibody against a specific modulation. A chromatin immunoprecipitation (ChIP) assay has also been reported to extract and quantify Ac-H3 from a cellular system [125].

### HDAC colorimetric assay

In contrast to the above, a commercial HDAC-specific coupled assay kit is available that makes use of a chromogenic peptide substrate (proprietary *Color de Lys*® Substrate) containing a ε-acetylated lysine residue [126]. When an HDAC enzyme acts upon the substrate and the sidechain of a ε-acetylated lysine residue is deacetylated, it becomes susceptible to further degradation by an enzyme in the developer reagent (proprietary *Color de Lys*® Developer). The action of the enzyme within the developer reagent results in the release of a chromophore detected by measuring the absorbance of the reaction at 405 nm (Fig. 2a). Since this is a colorimetric-based assay, it is generally of low sensitivity and prone to optical interference.

### HDAC fluorometric assays

This is a specific commercial HDAC coupled assay kit but significantly more sensitive that the colorimetric version described above. It is based upon a similar principle to the colorimetric but it involves the replacement of the chromogenic group with one that is fluorogenic [127]. The peptide substrate (proprietary *Fluor de Lys*® Substrate) once undergone deacetylation by HDAC enzyme is acted upon by the developer reagent (proprietary *Fluor de Lys*® Developer) resulting in the release of 7-amino-4-methylcoumarin fluorophore which undergoes excitation at 360 nm and emits at 460 nm (Fig. 2b). Fluorogenic assays offer significant advantages over colorimetric assays, as they are more sensitive and less prone to optical interference from compounds and have been utilized extensively by researchers to evaluate HDAC inhibitors [128–130].

### HDAC luminescence assays

This is another commercial coupled assay kit similar to those described above (colorimetric and fluorometric) but makes use of specific amino-luciferin labelled ε-acetylated lysine peptide substrates for HDAC Class I/II enzymes (Promega Corp. HDAC-Glo™ I/II Assays and Screening Systems) [131]. When the substrate undergoes deacetylation by the HDAC enzyme, the product becomes susceptible to the developer reagent and results in the release of amino-luciferin. This amino-luciferin is the substrate for a luciferase enzyme (also in the developer reagent) and yields a glow-type luminescence (Fig. 3). This assay has been validated and used to screen a natural product library [132].

### HDAC TR-FRET assay

This is a commercial assay kit (LANCE® *Ultra* TR-FRET) that uses a proprietary Europium chelate donor (W1024-Eu) linked to an anti-H3-K27(Ac) or anti-H3-K9(Ac) antibody, together with streptavidin-U*Light*™-acceptor. The available substrates for this are biotinylated Histone H3-K9(Ac) and Histone H3-K27(Ac) peptides. A TR-FRET signal is generated when the unmodified peptides are captured by the Europium-labelled antibody donor and streptavidin-U*Light*™ acceptor that brings the Europium-donor and U*Light*™-acceptor molecules into close proximity. Upon irradiation at 340 nm, the energy from the Europium-donor is transferred to the U*Light*™-acceptor, which in turn generates a signal at 665 nm (Fig. 4) [133].

A similar assay has been reported that is based on measuring the binding affinity of inhibitors rather than enzyme activity. As catalytically functional protein is not required, there is no requirement for a substrate and instead an Alexa Fluor® 647-labelled HDAC inhibitor is used as a tracer (acceptor in the TR-FRET assay). This can bind with specific HDAC enzymes tagged with GST in the presence of Europium anti-GST tag antibody (donor in the TR-FRET assay) and if the tracer is displaced by an appropriate compound, a decrease in signal would be observed [134].

## The demethylase target class and relevant screening compatible assays

The demethylase family of enzymes are responsible for the demethylation of lysine and arginine side chains in appropriate substrates (both histone and non-histone based) [135]. Specific examples of proteins in this class include lysine-specific demethylase (LSD) and the Jumonji C domain-containing histone demethylase (JHDM). This protein target class has been implicated in cancer [136], diabetes [137] and cardiovascular disease [138].

### LSD colorimetric assay

In this assay, the activity of human LSD1 makes use of dimethylated Histone H3-K4 peptide. This is a coupled assay in which the oxidative demethylation reaction catalysed by LSD1 results in the production of hydrogen peroxide (H_2_O_2_) [139–141]. This, in the presence of 3,5-dichloro-2-hydroxybenzenesulfonic acid and horseradish peroxidase (HRP), results in an absorbance change at 515 nm [142].

A commercial coupled assay kit is also available (Epigenase™ LSD1 Demethylase Activity/Inhibition Assay Kit) that makes use of a chromogenic peptide substrate. In the assay, microtiter plates coated with Histone H3-K4(Me_2_) LSD1 substrate are used, after which addition of LSD1 results in the removal of substrate methyl groups. After a wash step, the Histone H3-K4 demethylated product recognition takes place using a specific antibody and subsequently the colorimetric signal generated at 450 nm after addition of a proprietary detection mix (making use of the H_2_O_2_ or formaldehyde released as the by-product of LSD1 enzymatic reaction) [143].

### Jumonji C domain-containing histone demethylase fluorescence polarization assay

The crystal structure of histone demethylases have used in a structure-based drug design exercise to develop a substrate-derived inhibitor for Jumonji C domain-containing histone demethylase, termed methylstat [144]. This compound was shown to be active in vitro against isolated protein in a mass spectrometry (measuring H3-K9(Me_3_)) and in a cell-based HCS assay (measuring H3-K9(Me_3_)) using immunostaining with an anti-H3-K9(Me_3_) antibody. Modification of this compound with a fluorescent label has led to methylstat^fluor^, which has successfully been employed as a tracer in fluorescence polarization binding assay to monitor JHDM 1A activity [145].

### LSD fluorometric assay

This commercial assay kit works in a similar manner to the colorimetric kit described above but being fluorescence based. The assay is based on the multistep enzymatic reaction in which LSD1 first produces H_2_O_2_ during the demethylation of an Histone H3-K4(Me_2_) peptide. In the presence of HRP, H_2_O_2_ reacts with 10-acetyl-3,7-dihydroxyphenoxazine (also called Amplex Red) that results in the formation of Resorufin that can be quantified by fluorescence readout at excitation at 530 nm and emits at 590 nm [146]. A similar commercial kit is also available with an identical protocol but containing a proprietary Fluoro-Developer solution [147].

As an alternative, this commercial assay kit detects the formaldehyde released from the reaction of LSD1 when using an Histone H3-K4(Me_2_) protein. The formaldehyde released as the by-product of LSD1 reaction reacts with the proprietary detection reagent to generate a fluorescent signal with excitation at 410 nm and an emission at 480 nm [148]. Although the detecting reagent in the kit is proprietary, formaldehyde can be quantitated as the fluorescent condensation product 3,5,-diacetyl-1,4dihydrolutidine (DDL) which is formed with acetyl-acetone and ammonia in the Hantzsch reaction [149].

### LSD high content screening assay

This approach has been used to monitor the changes in H3-K27(Me_3_) and H3-K4(Me_3_) due to demethylase activity quantified in cell-based system using specific anti-H3-K27(Me_3_) and anti-H3-K4(Me_3_) antibodies. This approach was complemented with an in vitro assay using isolated lysine demethylase 6B (KDM6B) and chromatin immunoprecipitation (ChIP) assays using the same antibodies [150]. This panel of assays could be used to screen compounds in a low throughput manner and collectively they could provide information as to whether or not the compounds are LSD inhibitors.

### LSD mass spectrometry assay

This label-free approach has been used to measure LSD1 activity when using an Histone H3-K4(Me_2_) peptide substrate. The detection of demethylated product (H3-K4(Me)) was quantitatively determined by HPLC-MS [151]. As this is a low throughput assay, a relatively low number of compounds were screened.

This technique has also been used to monitor LSD2 activity using an Histone H3-K4(Me_2_) peptide substrate. The demethylation efficiency of LSD2 was estimated by mass spectrometry on the basis of detection of the product H3-K4(Me) peptide [152].

### LSD radioactive assay

This assay measures the release of radioactive formaldehyde from ^3^H-labelled methylated histone substrates when acted upon by LSD1 [153]. The radioactive formaldehyde is captured and separated from residual substrate and this assay is very sensitive and compatible for use with tissue and cell lysates [153]. However, it is limited by the method of radioactive substrate preparation and the formaldehyde detection method which requires the conversion of formaldehyde to DDL [154].

### LSD TR-FRET assay

This is a commercial assay kit (LANCE® *Ultra* TR-FRET) that works upon the same principle as shown above for the analogous assay for HDAC enzyme. In this case, the assay makes use of a biotinylated Histone H3-K4(Me) peptide substrate, with the unmodified peptide being captured by an Europium-labelled antibody as donor and U*Light*™-streptavidin that binds the peptide substrate [155].

The detection of H3-K27(Me_3_) in cell-based assay system has also been reported and the findings were further confirmed using alternative assay formats, namely AlphaLISA*®* and Western blot [156, 157].

## The histone methyltransferase (HMT) target class and relevant screening compatible assays

Histone methyltransferases (HMTs) enzymes catalyse the transfer of methyl groups to histone proteins and consequently, this can control or regulate DNA methylation through chromatin-dependent transcription repression or activation. Histone methylation serves in both epigenetic gene activation and silencing, thereby making it important to measure the activity or inhibition of HMTs and are implicated in cancer [158], HIV [159] and cardiovascular disease [160].

### HMT AlphaLISA^®^ assay

This is a commercial assay kit that detects changes in the levels of Histone H3-K79(Me_2_) protein [161]. The changes in the levels of Histone H3-K79(Me_2_) were performed by addition of anti-Histone H3 (C-terminal) AlphaLISA^®^ acceptor beads and biotinylated anti-dimethyl-H3-K79(Me_2_) antibody and streptavidin-donor beads.

### HMT fluorescence polarization assay

This is a generic methyltransferase assay that detects S-adenosylhomocysteine (SAH) product formation. The assay uses a highly specific immunodetection of nucleotide reaction products with the fluorescence polarization readout. This method requires an antibody that specifically binds SAH in the presence of excess S-adenosyl-L-methionine (SAM) and can differentiate on the basis of a single methyl group [162]. This assay has the advantage of being compatible with other enzymes of the same target class.

### HMT fluorometric assay

A coupled assay that relies upon the determination of SAM-dependent methyltransferase acting upon a H3 peptide. The SAH that is hydrolyzed by the coupling enzyme SAH hydrolase to homocysteine and adenosine. The free sulfhydryl group on a homocysteine molecule reacts with the maleimido form of the fluorophore, Thioglo1 forming a highly fluorescent conjugate with excitation at 382 nm and emission at 513 nm [163] and this method has been patented [164]. An alternative to Thioglo1 is 7-diethylamino-3-(4-maleimidophenyl)-4-methylcoumarin (CPM) which has been used to determine the activity of a number of methyltransferase enzymes [165].

### HMT high content screening assay

An ultra-high throughput screening assay (1536 wells) has been reported for determining the changes in H3-K27(Me_3_) in HeLa cells [166]. The assay quantifies the reduction in total H3-K27(Me_3_) using a specific antibody. The use of this assay in conjunction with a target-based assay for Enhancer of zeste homolog 2 (EZH2) histone-lysine N-methyltransferase enzyme enabled the assignment of any cellular activity to this specific target.

### HMT luminescence assay

This assay has been reported for histone methyltransferases in which the enzymes catalyse the transfer of a methyl group from SAM to a lysine amino group in a histone substrate resulting in the formation of SAH. The assay is novel in that the quantification of enzyme activity takes place via three coupled steps [167] and therefore is undesirable from a screening perspective.

### HMT radiometric assay

The activity of protein arginine methyltransferase 1 and 5 have been reported to make use of biotinylated peptides, ^3^H-SAM and streptavidin-coated SPA beads in a homogenous format that do not require any wash steps. Incorporation of radioactivity into the biotinylated peptides immobilized onto the SPA beads would lead to an increase in signal [168]. An analogous assay for *Neurospora crassa* Dim-H3-K9 methyltransferase that involves wash steps has also been reported which uses streptavidin microtiter plates coated with biotintinylated-H3K9 peptide substrate. Subsequently, the enzyme and ^3^H-SAM are added resulting in the transfer of the methyl groups to the target peptide. This brings the radioactive methyl group and scintillator in close proximity and an increase in signal [169]. This assay has also been applied to most other human methyltransferases [170].

## Histone acetyltransferase (HAT) assays

Histone acetyltransferase (HAT) enzymes catalyse the transfer of acetyl group from acetyl-CoA to histone proteins and are implicated in cancer [171], cardiovascular disease [172] and neurodegenerative disorders [173].

### Colorimetric assay

This is a commercial assay kit in which acetylation of a proprietary peptide substrate by all HAT enzymes releasing CoA-SH, which then serves as an essential coenzyme for producing NADH. The detection of NADH takes place spectrophotometrically at 440 nm upon it reacting with a soluble tetrazolium dye [174].

### ELISA

This is also a commercial assay kit to detect the presence of Histone H3-K4(Ac). In this assay, histone substrates are captured using Histone H3-coated antibody, followed by incubation with HAT enzymes allowing generation of product. Subsequent addition of a modification-specific primary antibody, anti-H3-K4(Ac) and secondary antibody coupled to HRP and a proprietary developing solution results in an increase in absorbance at 450 nm [175].

### Fluorometric assay

An assay for lysine acetyltransferase Rtt109 that upon transferring an acetyl group from acetyl-CoA to specific histone-lysine residues of its substrate results in the generation of CoA. The free thiol group of CoA reacts with the sulfhydryl-sensitive probe CPM to form a fluorescent adduct that is detected [176].

Another commercial assay kit that uses Histone H3 and Histone H4 N-terminal peptides as substrates. The HAT enzyme catalyses the transfer of acetyl groups from acetyl-CoA to the histone peptide thereby generating the acetylated peptide and CoA-SH. After stopping the reaction and addition of a developing solution, it reacts with the free sulfhydryl groups on the CoA-SH to give a fluorescent readout [177].

### Microfluidic mobility shift assay

This makes use of fluorescently labelled peptide substrates (derived from Histone H3 and Histone H4). Upon modification of the peptides, the substrate and product have a difference in charge and microfluidic electrophoresis allows their separation and quantification [178]. This assay was used to profile known and novel modulators of lysine acetyltransferase enzymes.

### Radiometric assay

In this assay, a synthetic biotinylated Histone H4-derived peptide acts as an HAT substrate [179, 180]. The enzyme acts upon [^14^C]acetyl-CoA and generates a radiolabelled peptide which is retained on streptavidin beads and subsequently counted in a liquid scintillation counter.

This assay makes use of radiolabelled [^3^H]-acetyl-CoA that is coated onto microtiter plates. Upon acetylation of lysine-rich Histone from calf thymus, the reaction is stopped and the signal counted using a scintillation counter [181].

### TR-FRET assay

This is a commercial assay kit that measures acetylation of biotinylated Histone H3K9 peptide. Upon acetylation of the peptide, it is captured by an Europium-labelled anti-H3-K9(Ac) antibody and *ULight®*-streptavidin which bring the Europium-donor and U*Light*™-acceptor molecules into close proximity and thus a TR-FRET signal is generated [182].

### The bromodomain target class and relevant screening compatible assays

Bromodomains are protein modules that bind to acetylated lysine residues and hence facilitate protein–protein interactions. Bromodomain-mediated interactions have key roles in transcriptional regulation and their dysfunction is implicated in a large number of diseases including cancer [183–185], atherosclerosis [186] and diabetes [187].

### AlphaScreen^®^ assay

This is an assay reporting the detection of BRD4 binding to Histone H4-K5(Ac) [188]. A biotinylated Histone H4-K5(Ac) substrate binds GST-bromodomain-containing protein 4 (GST-BRD4) and this complex binds a streptavidin-donor and glutathione-acceptor enabling them to come into close proximity, thereby yielding a signal.

In addition, interactions between His-BRD4 and biotinylated Histone H4-K4(Ac) have been reported that made use of streptavidin-donor beads and Ni-acceptor beads to enable the formation of the detection sandwich [189].

### Differential scanning fluorimetry assay

An assay has been reported that makes use of unlabelled BRD4 and SYPRO Orange Protein Gel Stain as a fluorescent probe [190]. It involved heat-induced protein denaturation which exposes hydrophobic surfaces that interact with SYPRO Orange, thereby increasing its fluorescence. The fluorescence gradually increases with increasing temperature and this data yields a melting temperature (*T*_m_) that is represented by an inflection point on the curve. Interactions between the protein and a ligand increases protein stability, leading to an increase in *T*_m_ and used to predict the *K*_d_ of compounds that were tested. This is also available commercially as the BromoMELT™ kit [191].

### Fluorescence polarization assay

A fluorescence polarization assay has been reported that makes use of a fluorescent BODIPY labelled tracer (BODIPY-BI2536) binding to BRD4 [190]. When the BRD4/BODIPY-BI2536 complex is in the presence of a compound that can displace the tracer from BRD4, a reduction in signal is observed. This assay was validated using a number of reference compounds.

### TR-FRET assay

This is a commercial assay kit that allows the characterization of BRD4/peptide interaction. The donor consists of BRD4 bromodomain 1 peptide labelled with Europium chelate. A biotinylated peptide containing the target acetylated lysine serves as the ligand for BRD4 bromodomain 1. APC-labelled avidin can bind with high affinity to the peptide substrate via the biotin moiety and serves as the acceptor in the assay [192] and any compound that displaces the complex will result in a decrease in signal.

## Conclusions

The body of evidence implicating epigenetic proteins in regulating biological processes and their dysfunction being the cause of various diseases is continuously increasing [119, 193]. This has led to significant drug discovery research efforts and the Food and Drug Administration (FDA) approval of a number of drugs and an even larger number of compounds being evaluated in clinical trials [119, 194, 195]. This trajectory of epigenetic drug discovery research is similar to that for the kinase protein target class after they were discovered and it is anticipated that many of the lessons learnt will apply to the epigenetic targets. For example, despite the initial concerns that selective kinase drugs may not be achievable due to their common ATP binding sites, Lapatinib has been shown to be a highly selective receptor tyrosine kinase inhibitor and has been approved for clinical use [196]. Another important example of a kinase inhibitor is Palbociclib which is an oral, reversible, selective, small-molecule inhibitor of cyclin-dependent kinase (CDK) 4 and CDK6 for the treatment of cancer. Early drug discovery efforts for the CDKs did not yield selective inhibitors and as a consequence they were considered as being intractable for inhibition by small molecules [197]. The research efforts in the kinase area have also led to the development of powerful assay methodologies to monitor their activity and currently more than 25 kinase assay formats are available [26, 198].

An additional therapeutic approach is to use combinations of drugs, e.g. kinase and epigenetic drugs [199, 200]. However, these may be associated with side effects and the possible origins of some of these for epigenetic drugs such as panobinostat have been elucidated using thermal proteome profiling [201]. This approach made use of a cellular thermal shift assay in conjunction with mass spectrometry-based proteomics. In this assay, HepG2 cells were incubated with panobinostat and subsequently those targets that were bound to the compound were identified. The bound proteins included HDAC targets as well as phenylalanine hydroxylase. The loss of function due to phenylalanine hydroxylase inhibition would be expected to increase phenylalanine levels in plasma and eventually decrease tyrosine levels. This could explain the symptoms mimicking hypothyroidism, a common panobinostat side effect.

It is anticipated that the next generation of epigenetic drugs will have reduced toxicity and improved efficacy as these are the major causes of attrition in drug discovery in the clinic [202, 203]. In order to identify the toxicity and efficacy problems early in the drug discovery workflow, there is an urgent need to establish more predictive and physiologically relevant assays such as those that use three-dimensional organoid cultures to study human disease processes [53, 204–206]. It can be difficult to interpret the screening output from such assays as the observations are likely to originate from compounds modulating a variety of cellular processes. This ambiguity can be reduced significantly with the cellular thermal shift assay (CETSA) that enables target engagement by compounds to be studied [207]. Other assay techniques that are now being used more commonly include advanced mass spectrometry [208–210] and when applied in conjunction with advanced image analysis of clinical samples, exquisite detail of cellular processes can be deciphered as shown in the case of the in situ detection of topoisomerase [211].

This review provides details of the current status of the assays that are available to monitor the activity of epigenetic targets. Since there are a number of assays that can be developed for any given target, it is prudent to develop a panel of assays as these can be used to confirm the observations across different readouts. This is illustrated methodically for the lysine demethylases that make use of target-based assays, crystallography and cell-based assays and should serve as a template for epigenetic drug discovery research [212].

## Abbreviations

Acetyl-CoA: Acetyl-coenzyme A
ADME: Absorption, Distribution, Metabolism and Excretion
APC: Allophycocyanin
BODIPY: 4,4-Difluoro-4-bora-3a,4a-diaza-s-indacene
BRD4: Bromodomain-containing protein 4
CDK: Cyclin-dependent kinase
CETSA: Cellular thermal shift assay
CoA-SH: Coenzyme A
CPM: 7-Diethylamino-3-(4-maleimidophenyl)-4-methylcoumarin)
DLL: 3,5,-Diacetyl-1,4dihydrolutidine
ELISA: Enzyme-linked immunosorbent assay
EZH2: Enhancer of zeste homolog 2
FDA: Food and Drug Administration
GST: Glutathione S-transferase
H2L: Hit-to-lead
H_2_O_2_: Hydrogen peroxide
HAT: Histone acetyltransferase
HCS: High content screening
HDAC: Histone deacetylase
His: Histidine
HMT: Histone methyltransferase
HRP: Horseradish peroxidase
HTS: High throughput screening
JHDM: Jumonji C domain-containing histone demethylase
LSD: Lysine-specific demethylase
mM: Millimolar
NADH: Nicotinamide adenine dinucleotide
Ni: Nickel
nm: Nanometer
SAH: S-Adenosylhomocysteine
SAM: S-Adenosyl-L-methionine
SAR: Structure-activity relationship
SPA: Scintillation proximity assay
*T*_m_: Melting temperature
TR-FRET: Time-resolved fluorescence resonance energy transfer
